# ATP-dependent helicase activity is dispensable for the physiological functions of Recql4

**DOI:** 10.1371/journal.pgen.1008266

**Published:** 2019-07-05

**Authors:** Wilson Castillo-Tandazo, Monique F. Smeets, Vincent Murphy, Rui Liu, Charlotte Hodson, Jörg Heierhorst, Andrew J. Deans, Carl R. Walkley

**Affiliations:** 1 St. Vincent’s Institute of Medical Research, Fitzroy, VIC, Australia; 2 Department of Medicine, St. Vincent’s Hospital, The University of Melbourne, Fitzroy, VIC, Australia; 3 Mary MacKillop Institute for Health Research, Australian Catholic University, Melbourne, VIC, Australia; University of Washington, UNITED STATES

## Abstract

Rothmund-Thomson syndrome (RTS) is a rare autosomal recessive disorder characterized by skin rash (poikiloderma), skeletal dysplasia, small stature, juvenile cataracts, sparse or absent hair, and predisposition to specific malignancies such as osteosarcoma and hematological neoplasms. RTS is caused by germ-line mutations in *RECQL4*, a RecQ helicase family member. *In vitro* studies have identified functions for the ATP-dependent helicase of RECQL4. However, its specific role *in vivo* remains unclear. To determine the physiological requirement and the biological functions of Recql4 helicase activity, we generated mice with an ATP-binding-deficient knock-in mutation (*Recql4*^K525A^). *Recql4*^*K525A/K525A*^ mice were strikingly normal in terms of embryonic development, body weight, hematopoiesis, B and T cell development, and physiological DNA damage repair. However, mice bearing two distinct truncating mutations *Recql4*^G522Efs^ and *Recql4*^R347*^, that abolished not only the helicase but also the C-terminal domain, developed a profound bone marrow failure and decrease in survival similar to a *Recql4* null allele. These results demonstrate that the ATP-dependent helicase activity of Recql4 is not essential for its physiological functions and that other domains might contribute to this phenotype. Future studies need to be performed to elucidate the complex interactions of RECQL4 domains and its contribution to the development of RTS.

## Author summary

DNA helicases unwind double-stranded nucleic acids using energy from ATP to access genetic information during cell replication. In humans, several families of helicases have been described and one of particular importance is the RecQ family, where mutations in three of five members cause human disease. RECQL4 is a member of this family and its mutation results in Rothmund-Thomson syndrome (RTS). Prior studies have shown that defects in the helicase region of RECQL4 may contribute to the disease, but no studies have specifically assessed the biological effects of its absence in a whole animal model. In this study, we generated a mouse model with a specific point mutation resulting in a helicase-inactive Recql4 protein. We found that an absence of ATP-dependent helicase activity does not perturb the physiological functions of Recql4 with the homozygous mutants being normal. In contrast, when we assessed point mutations that generate protein truncations these were pathogenic. Our results suggest that the helicase function of Recql4 is not essential for its physiological functions and that other domains of this protein might account for its functions in diseases such as RTS.

## Introduction

Rothmund-Thomson syndrome (RTS) (OMIM #268400) is a rare autosomal recessive disorder characterized by skin rash (poikiloderma), skeletal dysplasia, small stature, sparse or absent hair, gastrointestinal complications, and high predisposition to specific malignancies such as osteosarcoma (OS) and hematological neoplasms [1, 2]. RTS and the related RAPADILINO and Baller-Gerold syndromes are associated with damaging germ-line mutations in *RECQL4* [3–5]. The majority of *RECQL4* mutations are located in its helicase domain, yet the physiological role of this domain remains unclear [6].

Helicases are enzymes that unwind double-stranded or more complex DNA and RNA structures using energy from ATP hydrolysis. The unwinding of double-stranded DNA (dsDNA) is necessary to allow access to the DNA during replication, repair, recombination and transcription [7]. In humans, five members of the RecQ helicase family have been identified: *RECQL1*, *RECQL4*, *RECQL5*, *BLM* and *WRN*. Mutations in three are associated with syndromes that present with premature aging and cancer-predisposition: *WRN* in Werner’s syndrome, *BLM* in Bloom’s syndrome, and *RECQL4* in RTS [8, 9]. RecQ-family helicases contain three highly conserved protein domains: an archetypal helicase domain, which contains seven conserved motifs that couple ATP hydrolysis to dsDNA strand separation [8, 9]; the RecQ C-terminal (RQC) domain, which features a beta-hairpin motif, a winged-helix domain and a zinc-binding motif, for intervention in the binding of G quadruplex DNA and stabilization of DNA structures [10]; and the Helicase-and-ribonuclease-D-like C-terminal (HRDC) domain, which promotes stable DNA binding [11]. RECQL4 differs from the other family members as it has no HRDC domain and lacks a structurally conserved RQC domain. Instead, it contains the structurally unique domain called RecQ4-Zn^2+^-binding domain (R4ZBD) and, importantly, an N-terminal region of homology with the *S*. *cerevisiae* DNA replication initiation factor Sld2 [12, 13]. Sld2 is an essential protein required for activation of replication origins in yeast [14], and RECQL4 is the putative mammalian homologue.

The human *RECQL4* gene is located at the long arm of chromosome eight (8q24.3) and consists of 21 exons and 13 relatively short introns (<100 bp in length), yielding a full-length transcript of 3,627bp [3]. Mutations in the *RECQL4* gene have been found in the majority of RTS patients, and also in the related RAPADILINO and Baller-Gerold Syndromes [1, 6]. Most mutations are either nonsense or frameshift mutations and are predicted to create truncated proteins [6]. Over half of these impact the reading frame at exons 8–14 causing abnormal translation and or truncation of the RECQL4 protein with the presumed loss of DNA helicase function [6]. Very few mutations are located in the N-terminal Sld2 region, and those that are reported are primarily silent or missense [6]. This finding has led to the hypothesis that the N-terminal domain is critical for organismal viability, and that inactivation of the helicase function is a critical effect of the mutation spectrum that is found in RTS.

RECQL4 has been well characterized biochemically in terms of its single-stranded DNA (ssDNA) binding, ATP hydrolysis and DNA unwinding ability [15–17]. *In vitro*, RECQL4 ATPase and helicase activity is completely abolished by point mutations in the canonical Walker A and B motifs [18]. However, helicase-dead mutants can rescue cellular lethality in RECQL4-deficient DT40 chicken cells and murine hematopoietic cells *in vitro* [19, 20]. In addition, mutations that affect the helicase domain not only affect its activity but can also lead to protein truncation or unstable proteins, which does not allow the specific assessment of the physiological requirement of the ATP-dependent helicase RECQL4 in isolation. To understand the biological functions of RECQL4 helicase activity in a whole animal context, we generated a mouse model with a knock-in point mutation that specifically abolishes its ATP-dependent helicase activity (*Recql4*^*K525A*^) and compared this to two different truncating mutations *Recql4*^G522Efs^ and *Recql4*^R347*^. Here we show that mice with a specific deficiency in Recql4 helicase activity are strikingly normal, in contrast to pathogenic effects of truncating mutations that remove the entire helicase domain and downstream part of the protein.

## Results

### The *Recql4*^*K525A*^ mutation lacks ATP-dependent helicase activity

**Fig 1:**
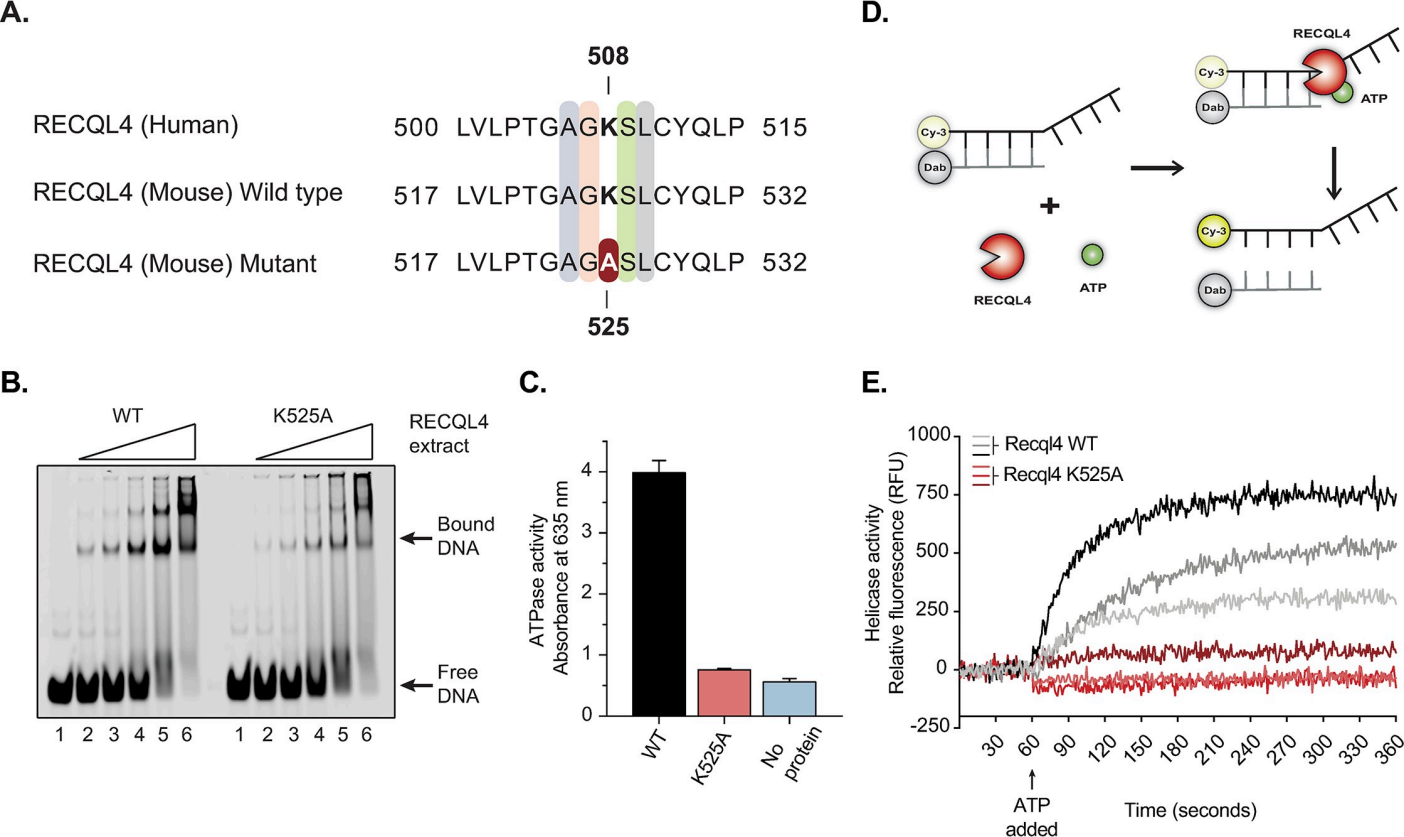
The *Recql4*^*K525A*^ mutation results in a biochemically inactive helicase protein. (A) Sequence homology between human and mouse RECQL4 showing the highly conserved amino acids between species. The helicase-dead mutation was achieved by replacing lysine by alanine in the 525 position in the mouse (508 in humans). (B) Electrophoretic-mobility shift assay (EMSA) comparing the DNA binding of WT versus K525A mutant Recql4. Lanes 1–6 show serial dilutions of Recql4 protein ranging from 19nM to 300nM bound to 25nM ssDNA oligo XOm1 conjugated to IRDye680. (C) *In vitro* ATPase assay of WT versus K525A mutant was obtained by measuring the absorbance at 635 nm. Proteins were assayed at 115nM in the presence of 1mM ATP and DNA in assay buffer. (D) Schematic representation of the fluorescence-based helicase assay. The dsDNA substrate with a ssDNA loading site is attached to a fluorescent donor (Cy-3) and a quencher acceptor (Dab). In the presence of ATP, the helicase (Recql4) is activated and separates the two complementary strands releasing the fluorescent probe. (E) Helicase assay of WT versus K525A mutant. After recording baseline fluorescence for 60 seconds, ATP was added, and helicase activity was measured in relative fluorescence units (RFU) for three different concentrations of Recql4 protein.

We generated full-length wild-type mouse Recql4 protein, along with a K525A variant. The alanine substitution replaced a critical lysine, present in all Walker A motif-containing ATPases, that is necessary for ATP hydrolysis and corresponds to a previously analyzed human RECQL4 K508A mutation that lacks ATP-dependent helicase activity [15, 21] (Fig 1A). To biochemically characterize an ATPase deficient mutation in murine Recql4, we established *in vitro* assays for Recql4 function. Both WT and K525A proteins had equivalent affinity for ssDNA binding, using an electrophoretic-mobility shift assay (EMSA) (Fig 1B). In ATPase assays, the WT protein hydrolyzed ATP in a DNA-dependent manner whereas Recql4^K525A^ showed no activity above background levels (Fig 1C). Finally, we tested the ability of these recombinant proteins to unwind DNA, using a dsDNA substrate with a ssDNA loading site. If ATP-dependent helicase activity is present, the fluorophore-labeled strand is released from the quencher-labeled complementary strand providing a real-time fluorescent readout of unwinding activity (Fig 1D). Robust ATP-dependent unwinding of the substrate was observed when using WT-Recql4 protein, whereas no change in fluorescence was observed using the Recql4^K525A^ mutant consistent with an inability to unwind DNA (Fig 1E). Together, our results demonstrate that the Recql4^K525A^ protein is helicase and ATPase dead, despite equivalent protein stability and DNA binding properties.

### Generation and validation of *Recql4* helicase-dead mutant mice

**Fig 2:**
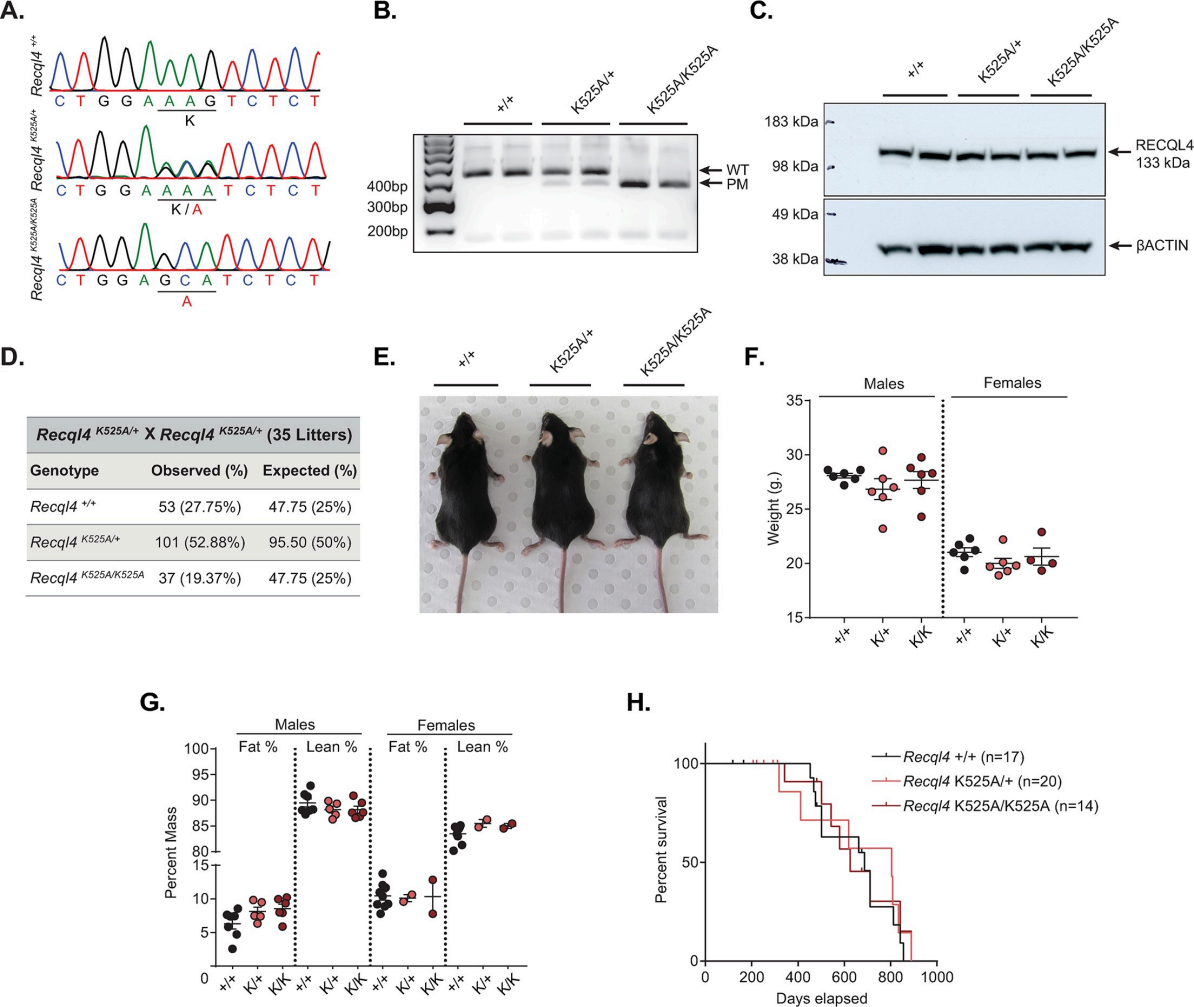
Homozygous and heterozygous helicase-dead mice are viable, have normal body weight and composition, and normal long-term survival. (A) Sequencing traces of the WT, heterozygous and homozygous K525A mutation. Altered nucleotide and amino acid changes are indicated above the sequence trace. (B) Genomic DNA PCR of *Recql4*^*+/+*^, *Recql4*^*K52A/+*^, *Recql4*^*K525A/K525A*^. 2 different mice per genotype. (C) Western blot of thymocyte lysates from *Recql4*^*+/+*^, *Recql4*^*K52A/+*^, *Recql4*^*K525A/K525A*^ 10 weeks old mice probed with anti-Recql4 (clone 3B10; top). The same blot re-probed with anti-β-Actin (bottom). (D) Breeding data from 35 litters of *Recql4*^*K52A/+*^ intercrosses. Observed and expected mendelian rates of the indicated genotypes are shown. No statistical significance was achieved. (E) Representative photograph of male *Recql4*^*+/+*^, *Recql4*^*K52A/+*^, *Recql4*^*K525A/K525A*^ mice. (F) Gross body weights of 10-week old male and female *Recql4*^*+/+*^, *Recql4*^*K52A/+*^, *Recql4*^*K525A/K525A*^ mice. (G) Echo-MRI analysis of fat and lean percentage at 10 weeks of age from male and female *Recql4*^*+/+*^, *Recql4*^*K52A/+*^, *Recql4*^*K525A/K525A*^ mice. (H) Kaplan-Meier survival plots of the indicated genotypes. K = K525A.

To understand the contribution of Recql4 helicase activity in the phenotypes of RTS, we generated an *in vivo* knock-in model of the K525A mutation. Sequencing of the *Recql4* locus in targeted mice confirmed the change in nucleotides encoding lysine (AAG) to alanine (GCA), and the resultant introduction of a unique *Msl*I restriction enzyme site in the mutant allele (Fig 2A). PCR amplification over the mutation site produces a 416 bp fragment in the wildtype that can cleaved to 361 bp (+55 bp) by *Msi*I when the mutation is present (Fig 2B). Finally, to determine the expression and stability of the mutant protein *in vivo* we generated a rat monoclonal antibody against the first 200aa from the N-terminal of murine Recql4 (clone 3B10). The K525A mutant protein has the same predicted molecular mass (~133kDa) as wild-type Recql4 and neither size nor abundance of the protein were affected by the mutation when assessed in thymocyte derived protein samples (Fig 2C). Taken together, these results demonstrate that the loss of helicase activity does not affect the expression or stability of the Recql4 protein *in vivo*.

### *Recql4* helicase-dead mice are viable, have normal body weight and composition and normal long-term survival

We observed a slightly sub-Mendelian ratio of homozygous *Recql4*^*K525A/K525A*^ animals at weaning from heterozygous breeding pairs, although this was not statistically significant by chi-squared test (p = 0.6255) (Fig 2D). Heterozygous and homozygous mice for the K525A mutation were viable and outwardly normal (Fig 2E). Further, we observed no difference across genotypes or sexes in 10-week old animals assessed for body weight and body composition (Fig 2F and 2G). Both male and female *Recql4*^*K525A/K525A*^ animals were fertile and able to breed, and there was no difference in the survival when comparing the *Recql4*^*K525A/K525A*^ and control genotypes using Kaplan Meier survival analysis (Fig 2H). Collectively these results show that, unlike the embryonic lethality of *Recql4* null alleles [20, 22], the Recql4^K525A^ protein supports normal development and adult homeostasis.

### Recql4 helicase activity is not required for normal hematopoiesis

**Fig 3:**
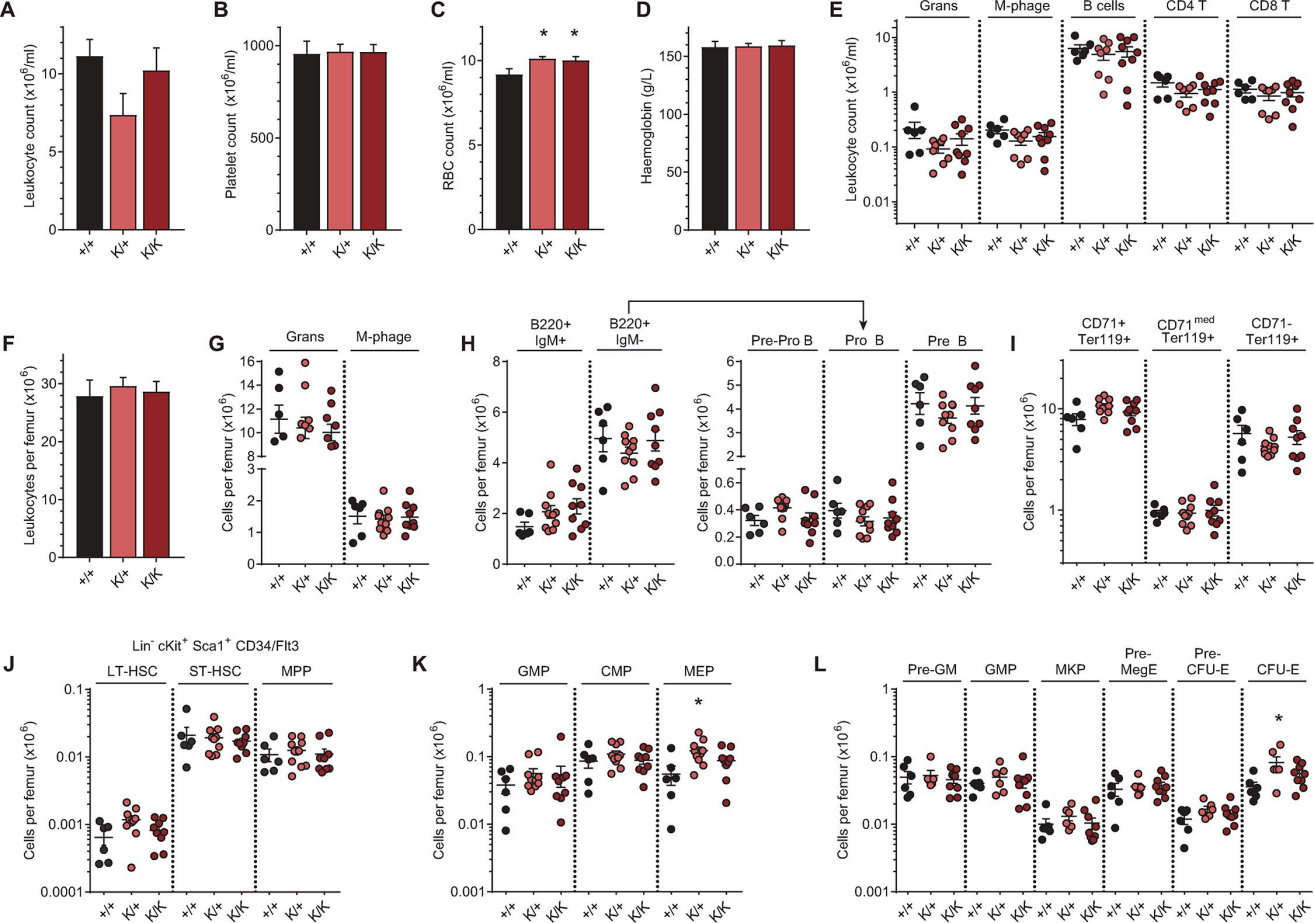
The ATP-dependent helicase function of Recql4 is not required for normal hematopoiesis *in vivo*. (A) Leukocyte counts in the PB of *Recql4*^*+/+*^, *Recql4*^*K52A/+*^, *Recql4*^*K525A/K525A*^ 10-week old mice. (B) Platelet count. (C) Red blood cell count. (D) Hemoglobin. (E) Numbers of granulocytes, macrophages, B cells, CD4 T cells, and CD8 T cells in PB. (F) Leukocyte counts in BM. (G) Absolute numbers of granulocytes and macrophages in BM. (H) Mature (B220^+^IgM^+^), immature (B220^+^IgM^-^) B lymphocytes, and subsets of immature B lymphocytes per femur. (I) Erythroid fractions based on CD71/Ter119 staining per femur. (J) HSC and primitive progenitors based on Lin^-^c-kit^+^Sca-1^+^CD34/Flt3 staining per femur. (K) Myeloid progenitor subpopulations in the BM. (L) Myeloid, erythroid and megakaryocyte progenitor frequency per femur. Data expressed as mean ± SEM, Student’s t test. *P<0.05; n≥6 per genotype. Experiments were independently executed on separate cohorts, with results pooled for presentation. K = K525A.

We previously reported that somatic deletion of *Recql4* resulted in a fully penetrant bone marrow (BM) failure [20]. To determine the role of helicase activity in hematopoiesis, we assessed cohorts of adult wild-type, *Recql4*^*K525A/+*^ and *Recql4*^*K525A/K525A*^ mice. Analysis of the peripheral blood (PB) revealed no changes in leukocyte or platelet numbers (Fig 3A and 3B). The absolute red blood cell counts were subtly increased in both the K525A/+ and K525A/K525A mutants, however, the hemoglobin levels were not changed compared to the WT (Fig 3C and 3D). Further analysis of the individual leukocyte subsets in the PB including granulocytes, macrophages, B lymphocytes, and CD4^+^ and CD8^+^ T lymphocytes revealed no significant differences in the proportions or absolute numbers of these populations (Fig 3E).

Within the BM, the total numbers of leukocytes were comparable across genotypes (Fig 3F). Similarly, granulocyte and macrophage numbers were similar (Fig 3G). Within the B-lymphoid populations (B220^+^IgM^-^), the Pre-B, Pro-B, and Pre-Pro-B subpopulations were all unaltered in *Recql4*^*K525A/+*^ and *Recql4*^*K52A/K525A*^ compared to WT littermates (Fig 3H), as were the erythroid subpopulations (Fig 3I). The frequencies and absolute numbers of the primitive hematopoietic stem cells (HSCs) and progenitors were also assessed. There were no major differences in the numbers of phenotypic long-term HSCs (LT-HSC), short-term HSCs (ST-HSC) or multipotent progenitor (MPP (Lin^-^ c-kit^+^ Sca-1^+^ CD34/Flt3)) fractions, nor in their myeloid committed subpopulations (Fig 3J–3L). There was an elevation of the phenotypic myelo-erythroid progenitor (MEP, Lin^-^c-kit^+^Sca-1^-^CD34/FcγRII) and colony forming unit-erythroid (CFU-E, Lin^-^LKS^-^CD41^-^FcγRII^-^CD150^-^CD105^+^) in the *Recql4*^*K525A/+*^ animals, however the absolute change was very small and not statistically significant in the *Recql4*^*K525A/K525A*^ mice. The basis for this mild elevation in RBC counts and committed erythroid progenitors is currently undefined. In summary, unlike for complete deletion of Recql4, the abrogation of Recql4 helicase activity does not substantively perturb hematopoiesis *in vivo*.

### ATP-dependent helicase function is not required for B and T cell development

**Fig 4:**
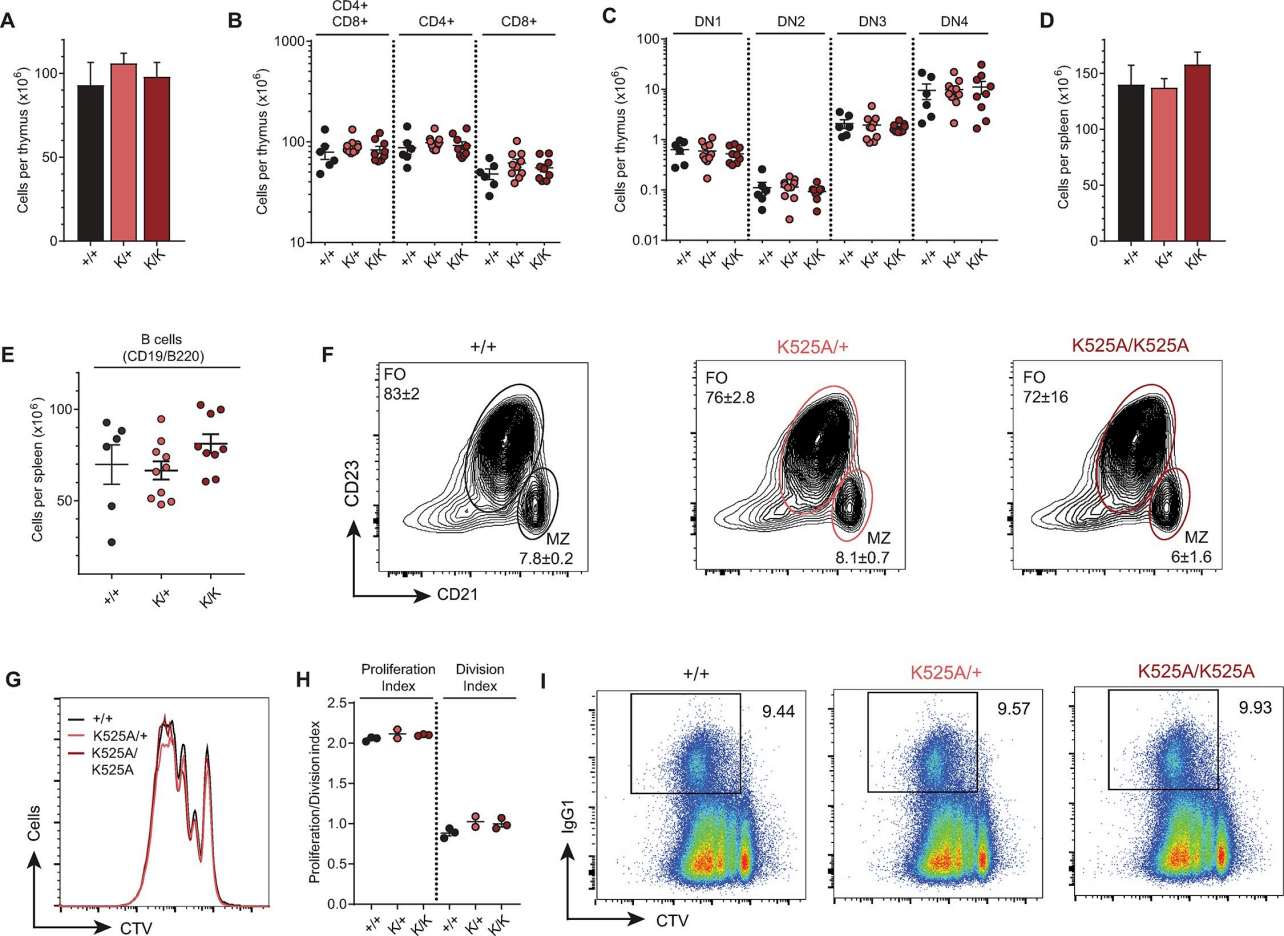
ATP-dependent helicase function of Recql4 is not required for B or T cell development. (A) Leukocyte counts in thymus. (B) Absolute numbers of double positive CD4/CD8, CD4^+^, and CD8^+^ T lymphocytes in thymus. (C) T cell progenitor populations (DN1-DN4). (D) Leukocyte counts in spleen. (E) B lymphocytes in spleen. (F) Representative (n = 2 mice per genotype) FACS plots of B220^+^CD19^+^ gated splenocytes stained for follicular (FO, CD23^hi^CD21^low^) and marginal zone (MZ, CD21^hi^CD23^low^) B cells. Numbers indicate cells x 10^6^ per spleen. (G) CellTrace Violet (CTV) dilution in purified B cells treated for 96 hours with 15μg/ml LPS + 10ng/ml IL4. Representative examples of n = 3 mice per genotype. (H) Quantification of cell division (average division per cell in culture) and proliferation (average division/per dividing cell in culture) indices. n = 3 mice per genotype. (I) *In vitro* class-switch recombination to IgG1 in purified B cells treated as in panel B. Representative examples of n = 2 mice per genotype. Data expressed as mean ± SEM, Student’s t test **(A-E)**. Experiments were independently executed on separate cohorts, with results pooled for presentation. K = K525A.

Our previous work demonstrated that the human helicase dead RECQL4 (K508A) was able to rescue *in vitro* B and T cell development from murine *Recql4*^*Δ/Δ*^ hematopoietic cells [20]. To determine if this was also the case *in vivo*, T and B cell development was assessed from thymocytes and splenocytes respectively in 10-week-old mutant and WT mice. There was no difference in thymus cellularity or in the numbers of double positive CD4^+^/CD8^+^ nor the mature single positive CD4^+^ and CD8^+^ cells (Fig 4A and 4B). Analysis of early intra-thymic progenitor cells (double negative DN1-4) found no difference in the *Recql4*^*K525A/K525A*^ compared to the WT littermates (Fig 4C). In the spleen there was no significant difference in the cellularity or number of mature B cells (Fig 4D and 4E). To determine the proportion of B cells in the follicular (FO) and marginal zone (MZ) of the white pulp of the spleen, we divided splenic cells with high expression of B220 and CD19 followed by analysis of CD21/CD23 expression (FO B cells: CD21^low^CD23^high^; MZ B cells: CD21^high^CD23^low^). No shift of B cells in the follicular or marginal zone compartments was apparent (Fig 4F). Therefore, consistent with the prior retroviral rescue data *in vitro*, the ATP-dependent helicase function of Recql4 is not required for B or T cell development and homeostasis in adult mice.

Given the role of RecQ helicases in repair of DNA damage [11], we sought to determine if there was a function for the ATP-dependent helicase activity of Recql4 during physiologically induced DNA damage occurring following B cell stimulation. We isolated mature B cells from WT, *Recql4*^*+/K525A*^ and *Recql4*^*K525A/K525A*^ spleens and stimulated them *in vitro* using bacterial lipopolysaccharide (LPS) and rmIL-4 to induce proliferation and immunoglobulin gene (*Ig*) class switch recombination. The proliferation of the cells in response to LPS was the same irrespective of Recql4 helicase status as measured by cell-trace violet dilution kinetics (Fig 4G and 4H). Additionally, the *Recql4*^*K525A/K525A*^ cells underwent normal class switching to IgG1 (Fig 4I). Therefore maturation, physiological activation induced proliferation, DNA damage and immunoglobulin rearrangement of B cells do not require the helicase activity of Recql4.

### The K525A mutation does not alter sensitivity to DNA damaging agents *in vitro*

**Fig 5:**
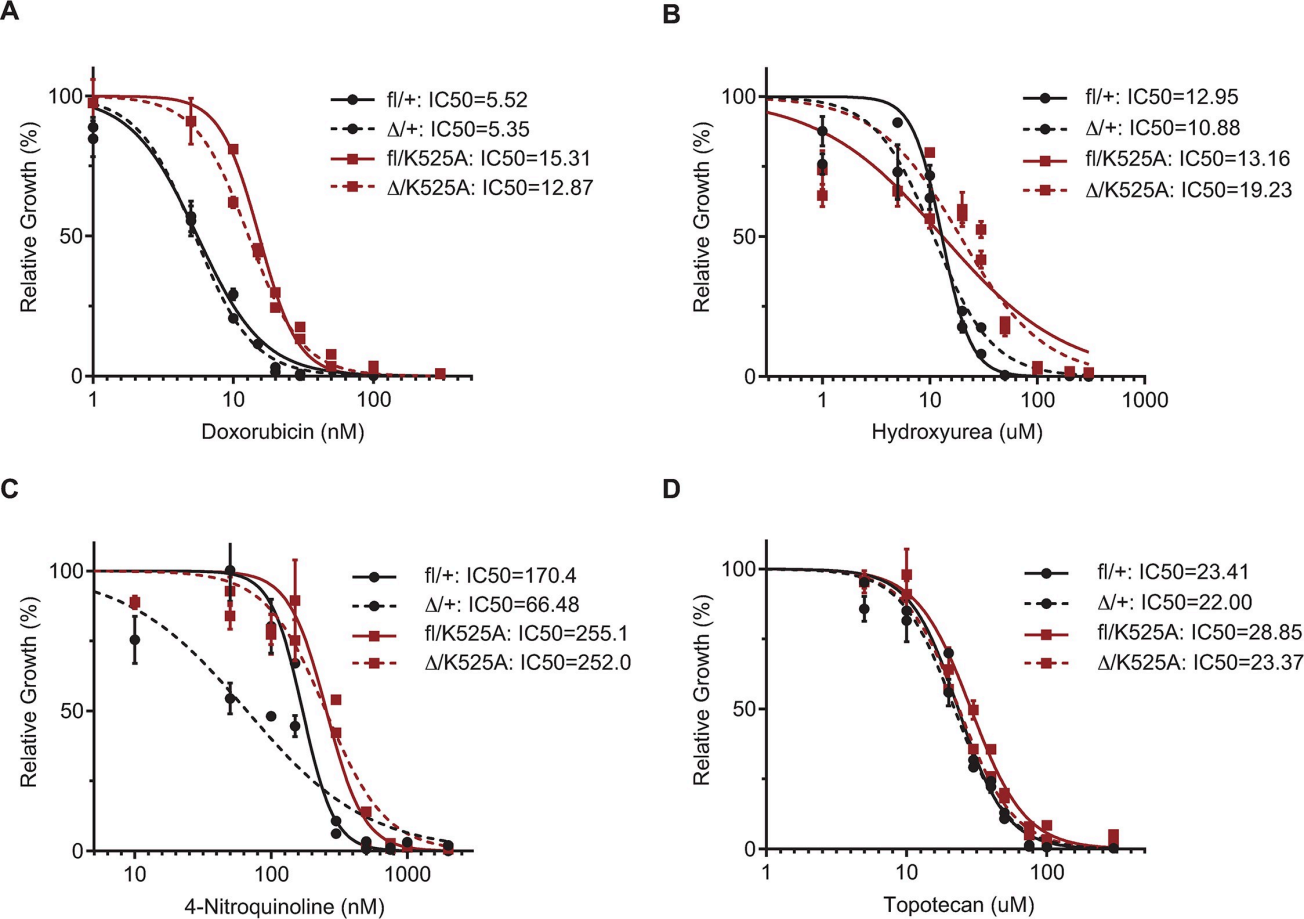
*In vitro* sensitivity to DNA damaging agents is not modified by helicase-dead mutation. Dose-response curves for relative growth rate of HoxB8 immortalized *R26*-CreER *Recql4*^*fl/K525A*^ (red) and *R26*-CreER *Recql4*^*fl/+*^ (black). Dotted lines represent cells expressing the K525 mutant only after the floxed allele was excised by tamoxifen addition. Cells were incubated for 48 hours with the following drugs: (A) Doxorubicin. (B) Hydroxyurea. (C) 4-Nitroquinoline. (D) Topotecan. The calculated IC_50_ values are stated for each genotype/treatment.

To further test the requirement for RecQ helicase activity we compared the response to DNA damaging agents *in vitro*. For these studies, we established GM-CSF dependent myeloid progenitor cell lines from *R26*-CreER *Recql4*^*fl/K525A*^ and *R26*-CreER *Recql4*^*fl/+*^ (control genotype) by immortalization with HoxB8 [23]. To allow analysis of the requirement for RecQ helicase activity, the cells were treated with tamoxifen for 4 days to activate Cre-mediated deletion of the loxP flanked wild-type Recql4 (*Recql4*^*fl*^) allele. This resulted in the cells either becoming Recql4 heterozygous (*Recql4*^*Δ/+*^) or expressing only the K525A mutant allele (*Recql4*^*Δ/K525A*^). Isogenic cells from each genotype (pre-treated with tamoxifen for 4 days or untreated) were seeded in 96-well plates and the response to four different genotoxic agents was assessed: doxorubicin (topoisomerase II inhibitor), hydroxyurea (ribonucleotide reductase inhibitor), 4-nitroquinoline (oxidative DNA damage) and topotecan (topoisomerase I inhibitor). Cell viability was measured after 48 hours using the CellTiter-Glo Luminescent assay. As shown in Fig 5A–5D, *R26*-CreER *Recql4*^*Δ/K525A*^ had a comparable IC_50_ to Doxorubicin, Hydroxyurea, 4-Nitroquinoline, and Topotecan as the non-tamoxifen treated isogenic controls (Figs 5A–5D and S3). Additionally, *R26*-CreER *Recql4*^*Δ/+*^ showed a similar IC_50_ to Doxorubicin, Hydroxyurea, and Topotecan, except for 4-Nitroquinoline, which exhibited a mildly increased resistance in the tamoxifen-treated cells in comparison to the non-tamoxifen treated controls. These results indicate that the role of Recql4 in non-physiological, pharmacologically induced DNA repair does not depend on its helicase activity.

### Truncating mutations, but not helicase inactivation, of Recql4 are pathogenic

**Fig 6:**
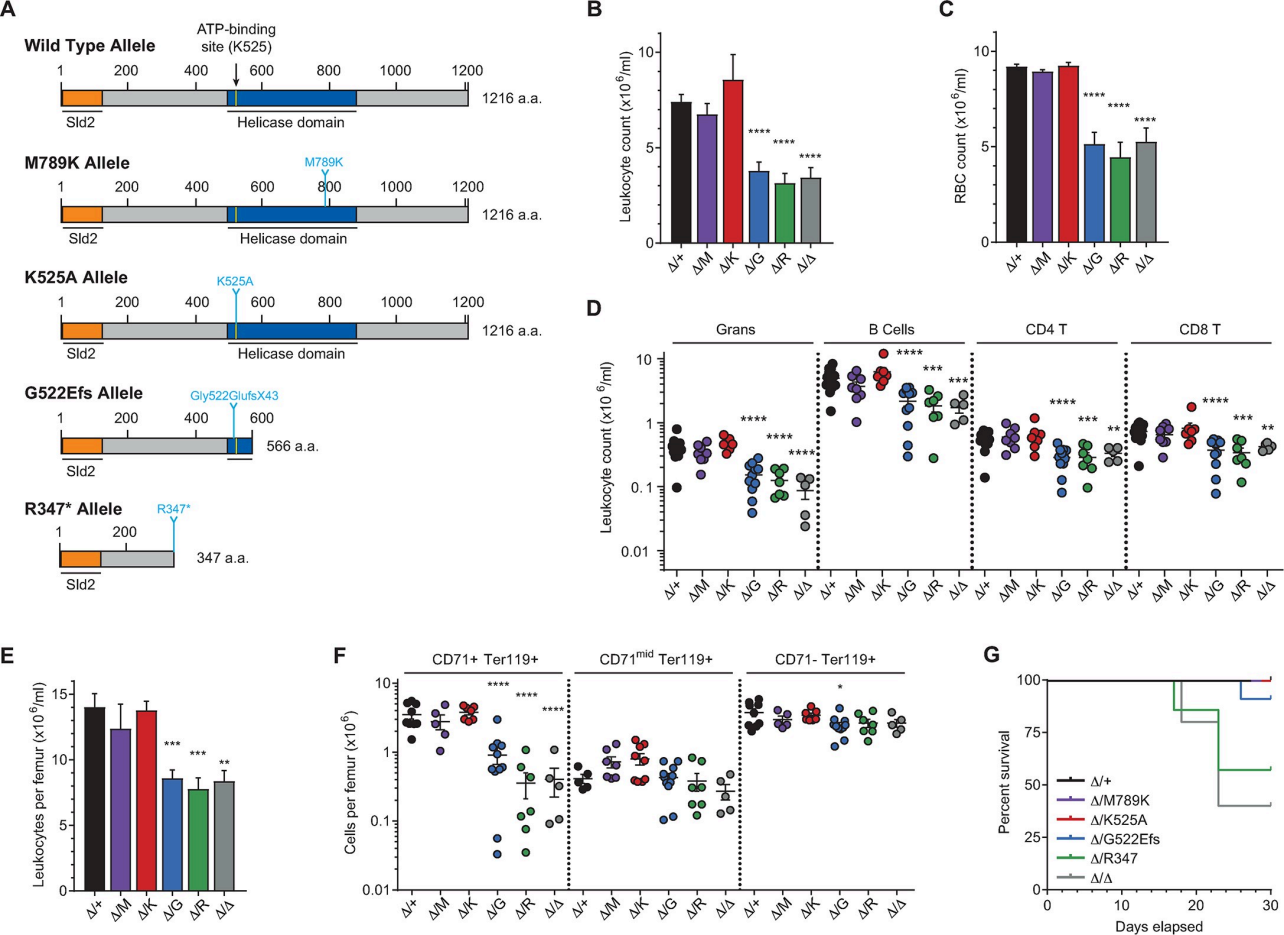
Truncating mutations, but not helicase inactivation, result in deleterious effects. (A) Schematic representation of the *R26-*CreER *Recql4*^*fl/PM*^ murine alleles used in this study. (B) Leukocyte counts in PB. (C) Red Blood Cell counts in PB. (D) Absolute numbers of leukocyte subsets in PB. (E) Leukocyte counts in BM. (F) Erythroid fractions based on CD71/Ter119 staining per femur. (G) Kaplan-Meier survival plots of the indicated genotypes in days on Tamoxifen food. All data are from day of sacrifice and expressed as mean ± SEM, Student’s t test. *P<0.05; **P<0.01; ***P<0.001; ****P<0.0001; n≥6 per genotype **(B-F)**. Experiments were independently executed on separate cohorts, with results pooled for presentation. M = M789K; K = K525A; G = G522Efs; R = R347*.

The results so far establish that the ATP-dependent helicase activity of Recql4 is not essential *in vivo*. To directly compare the effects of having a protein truncating/hypomorphic allele compared to a helicase-dead full-length Recql4 protein, we established three additional point mutant alleles (Fig 6A). The G522EfsX43 truncated mutation was generated as a co-incidental mutation during the Crispr/Cas9 mediate generation of the K525A allele and maps closely to the relatively common RTS associated mutations in human *RECQL4*, S523TfsX35 and C525AfsX33 [6]. We also identified an *N*-ethyl-*N*-nitrosourea (ENU) mutagenesis induced truncated mutation R347* (R355 in humans). Three RTS patients have been reported with p.Arg350GlyfsX21 mutations, mapping closely to this allele [6] (S2 Fig). A second ENU induced mutation, M789K mutation (V767 in human), was identified and used as a control for mutations within the *Recql4* locus as this mutation was predicted to be benign. We crossed all the individual point mutant alleles (M789K, K525A, G522Efs and R347*) to the *R26-*CreER *Recql4*^*fl/fl*^ line that we previously described and assessed the *R26-*CreER *Recql4*^*fl/PM*^ in parallel with the *R26-*CreER *Recql4*^*fl/+*^ and *R26-*CreER *Recql4*^*fl/fl*^ allele. [20]. At 8–10 weeks of age, mice were fed tamoxifen containing chow for 30 days to activate the Cre mediated deletion of the wild-type *Recql4* floxed allele broadly throughout the body. Using this experimental design, the tamoxifen treated *R26-*CreER *Recql4*^*fl/fl*^ animals (completely Recql4 deficient) developed fully penetrant BM failure [20]. The efficiency of *Recql4* deletion was confirmed by PCR for genomic recombination (S1 Fig).

Analysis of PB (Fig 6B) showed an approximately 50% reduction in leukocytes and erythrocytes in mice expressing the truncating mutations G522Efs and R347*, very similar to mice rendered null for Recql4 protein expression (*Recql4*^*Δ/Δ*^). The K525A and M789K mutation, as well as the heterozygous (*Recql4*^*fl/+*^) control, did not show any significant change in leukocyte or erythroid indices. In addition, analysis of individual lineages within the PB showed a similar pattern across granulocytes, B lymphocytes, and CD4^+^ and CD8^+^ T lymphocytes in the truncating and null mutant (Fig 6B–6D). When the BM was analyzed, leukocytes and erythroid precursors (CD71^+^Ter119^+^) from *R26*-CreER^ki/+^*Recql4*^*Δ/G522Efs*^ and *Recql4*^Δ/R347*^ mice showed a dramatic decrease, consistent with the BM failure phenotype we had previously described in *Recql4*^*Δ/Δ*^ (Fig 6E and 6F). The *Recql4*^*Δ/+*^ and K525A and M789K only expressing animals did not develop any phenotype after 30 days of treatment with tamoxifen and, whilst developing a pan-cytopenia in PB and BM, seven of eight G522Efs mice were still alive at the end of the treatment. In contrast, mice expressing the most severely truncating R347* allele developed a profound BM failure and three of five required euthanasia prior to 30 days of treatment, a phenotype similar to complete loss of Recql4 protein (Fig 6G). Collectively, these analyses establish that the ATP-dependent helicase is not required for the physiological functions of Recql4 *in vivo*, however mutations resulting in truncated protein products are deleterious.

## Discussion

The N-terminal Sld2-domain of RECQL4 protein is unique among RecQ family members and has been shown to be critical for the initiation of DNA replication in chicken, Drosophila, Xenopus and human cells [12, 17, 19, 24, 25], mostly likely through Sld2-domain dependent recruitment of the MCM10 and CTF4 factors to origins of replication [26]. The importance of the Sld2-domain to cell viability is reflected in the mutation spectrum detected in RTS patients–it is rarely mutated and always intact in at least one allele [6]. These findings implied that RTS and related disorders were caused by the loss of activity of the canonical ATP-dependent helicase domain, whose role in the replication initiation function is less clear. While initial *in vitro* studies using purified full-length human RECQL4 protein did not detect any unwinding potential on long DNA substrates [27], it was later shown that RECQL4 could unwind shorter duplex regions if a single strand-loading region was provided [16, 18]. This activity, together with the single-stranded DNA dependent ATP hydrolyzing activity, was lost in human Walker motif mutants RECQL4^K508A^ and ^D605A^. We now demonstrate that the murine Recql4^K525A^ mutant protein is able to bind DNA but cannot hydrolyze ATP nor unwind DNA substrates, confirming that the mutant is helicase-dead and the homologue of human RECQL4^K508A^. Unexpectedly, however, we found that this protein behaved comparably to wild-type Recql4 in supporting viability, fertility and normal physiological development of mice. No phenotypes or symptoms consistent with RTS were observed. Perhaps more surprising, was the normal response of Recql4^K525A/K525A^ cells in replication, and in their response to both physiologically or exogenously induced DNA damage.

The most highly conserved domain of the RecQ helicases is the ATPase core. By analogy to multiple other DNA helicases (reviewed in ref. [28]), it was assumed that the ATP-dependent helicase activity of RECQL4 is essential for normal cellular DNA metabolism. Several studies have reported findings consistent with this interpretation. Complementation experiments in *Drosophila* showed that the helicase-dead K898N mutant could not rescue the viability of RecQ4 null mutants [29]. Similarly, a helicase-inactive human D605A mutant could not restore replication of xRecql4 depleted cells in *Xenopus* egg extracts [12]. Murine studies to date have only assessed complete nulls or severe hypomorphic alleles. The deletion of the exons that precede the helicase domain (exons 5–8) resulted in embryonic lethality by day 3–6 and a significantly truncated protein with no helicase or C-terminal domain [22]. When the entire helicase domain was deleted (exons 9–13; in-frame deletion), mice were viable but showed high rates of perinatal lethality [30] and a truncated protein product of 480aa was predicted. With the aim to maintain an intact protein, a study replaced the last helicase coding exon (13) with a neomycin cassette. 95% of animals died within 2 weeks after birth and a substantial number of short transcripts covering exon 1 to 12 were reported, encoding potentially truncated products [31]. Since all these models effectively create proteins that lack the helicase and C-terminal domain, it is unclear if the observed phenotype could be attributed to the absence of the helicase domain only. Our study demonstrates that mice carrying the K525A helicase-dead mutation, with a stable full-length Recql4 protein, were viable and fertile with no apparent phenotype. The *in vivo* studies reported herein support a conclusion that the ATP-dependent helicase activity of Recql4 is not essential for replication or viability and that other domains account for these functions.

A range of prior *in vitro* studies have pointed to the importance of the N-terminal Sld2-like region. Lethality of *RECQL4*-depleted chicken cells was rescued by expression of the N-terminal region only [19]. In addition, the N-terminal domain of RECQL4 was shown to physically interact with several proteins involved in DNA replication in *Xenopus laevis* and human cell extracts [32, 33]. In our *in vivo* experiments however, we have shown that one copy of the N-terminal region alone (R347* or G522Efs) is insufficient for viability, indicating that a certain level of expression or localization of full-length Recql4 protein is required even if it has no ATP-ase or unwinding capacity.

The high frequency of chromosome abnormalities found in cells from RTS patients and the increased cancer incidence rates, suggest that RECQL4 may have a role in maintaining genome stability through DNA repair [2]. Prior studies have attributed several DNA repair functions of RECQL4 to its ATP-dependent helicase region, but they also have noted participation of the N-terminal region. Lu et al. found that a helicase-dead K508M could not rescue the loss of DNA end resection and homologous recombination (HR) repair after RECQL4 siRNA knock down, suggesting that the ATP-dependent helicase function of RECQL4 is involved in HR [34]. However, they also showed that it is the N-terminus of RECQL4 that physically interacts with MRN and CtIP [35]. In a similar fashion, a role for RECQL4 in non-homologous end joining (NHEJ) was linked to its interaction with the Ku70-80 by the N-terminal domain [36]. RECQL4 deficiency has been associated with modulation of core proteins involved in base excision repair (BER) such as POLB, FEN1, and APE1. The latter has shown to specifically interact with the N-terminal region of RECQL4 [37]. Herein, we observed no differences in the sensitivity of helicase-dead mutant cells compared to WT cells in response to various kinds of DNA damage (NHEJ, MMEJ, and HR for doxorubicin, BER for hydroxyurea, NER and BER for 4-nitroquinoline, and BER/SSBR for topotecan). Taken together there was no evidence for a role for the ATP-dependent function of Recql4 in the repair of pathological environmentally induced DNA damage.

A recent study showed that RECQL4-depleted U2OS cells were also deficient in ATM dependent checkpoint activation in response to drug induced DNA DSBs. Complementation assays using helicase-inactive point mutants in Walker A (K508G) or Walker B motif (D605A and E606A) further indicated that this was the result of a lack of helicase activity [38]. The ATM pathway plays an equally important role in the physiological processes of DSB repair and recombination, such as V(D)J recombination in T cell development and class switch recombination in B cell activation [39–41]. In our *in vivo* analysis we did not detect any defect in T cell maturation at the CD4^+^/CD8^+^ double positive to CD4^+^ and CD8^+^ single positive transition or any earlier stage, nor did the mice develop any T cell lymphomas as a result of chromosomal anomalies. In addition, *in vitro* B cell activation and class switch recombination in helicase-dead splenic B lymphocytes was indistinguishable from that in WT cells, arguing that the helicase activity is not required for either physiological checkpoint activation or DNA damage repair.

RTS patients, however, usually present with compound heterozygous mutations. It was reported that in 46% of RTS patients compound heterozygous mutations were present in the *RECQL4* gene [6]. The majority of these mutations affect the helicase and C-terminal region and are predicted to create truncated proteins caused by an early stop codon, frameshift, or mis-splicing [6]. The phenotypes in RTS patients, although grossly similar, can vary widely in severity. The relatively common C525AfsX33 (12 alleles) for example, has been found in all three syndromes (RTS, RAPADILINO and Baller-Gerold) and no single mutation has been assigned to a specific set of clinical features. In our study we demonstrate that mice carrying a sole truncating mutation (G522Efs and R347*) presented with a BM failure reminiscent of the *Recql4* null. This was not seen in the helicase-dead K525A, the M789K or the WT heterozygous null mice. Furthermore, when we assessed BM, PB, thymus, and spleen from heterozygous and homozygous K525A helicase-dead mutants, we did not find any significant change. A similar observation was made for the human WRN helicase. A naturally occurring single nucleotide polymorphism (R834C) was shown to have less than 10% of the WT helicase activity, but normal exonuclease activity. None of the heterozygous or homozygous carriers of this mutation developed Werner Syndrome (as defined by the clinical phenotype), clearly separating the WRN helicase function from other WRN functions [42]. Our findings demonstrate that helicase activity of Recql4 is also not required *in vivo* in mammals.

Collectively, this study has demonstrated the ATP-dependent helicase activity of Recql4 is not physiologically essential for murine embryonic development or adult homeostasis, cellular replication and physiological DNA damage repair. However, mutations that create truncating proteins are not tolerated. Further studies will have to be performed to elucidate the complex interactions of Recql4 mutations, their role in RTS and the contribution of the individual Recql4 domains to its normal physiological function.

## Materials and methods

### Ethics statement

All animal experiments were approved by the Animal Ethics Committee, St. Vincent’s Hospital, Melbourne, Australia (#007/14 and 011/15). Animals were euthanized by CO_2_ asphyxiation or cervical dislocation.

### Recql4 cloning, expression and purification

The full-length WT and K525A mutant codon optimized cDNA sequence of the mouse Recql4 containing 3xFLAG tag at the C-terminus were cloned into vector pFL-EGFP and transferred to the Multibac expression system to generate baculovirus [43]. Baculovirus infected High 5 cells were resuspended in TNG buffer (20mM TEA pH7.5, 150mM NaCl, 10% glycerol, 1mM EDTA, 1x mammalian protease inhibitors (Sigma-Aldrich) and 1mM DTT. Mixtures were sonicated three times for 30 seconds on ice. Lysates were clarified by centrifugation at 50K x *g* 30 minutes. Anti-Flag M2–Affinity Gel (Sigma-Aldrich) resin was added and incubated for 60 minutes. Resin was extensively washed with TNG (without PI or EDTA), then washed overnight in TNG containing Benzonase nuclease (Sigma-Aldrich). Further washes were performed to remove the nuclease. Subsequently Recql4 was eluted with 100ug/ml Flag peptide in TNG.

### Recql4 helicase assay

Helicase assays were performed according to Kaiser et al [18]. 80μl reactions containing 0.5μM protein and 50nM of a 15nt 3’-overhang (OH) DNA substrate in assay buffer (20mM HEPES pH 8.0, 10mM NaCl, 5% Glycerol, 1mM MgCl_2_, 0.5mM TCEP) were assayed in an EnSpire 2300 microplate reader (Perkin Elmer) at 25°C. The DNA substrate (T3-Cy3 annealed to B1-Dab) contained a 3′-15nt polyT ssDNA loading site and a 15nt dsDNA part with a generic sequence. After recording baseline fluorescence for 60s (Excitation 530nm / Emission 580nm), the helicase reaction was initiated by adding ATP to a final concentration of 1.25mM and the increasing Cy3-fluorescence as the quencher-labelled bottom-DNA strand is separated from the Cy3-labelled top-DNA strand, was recorded for 5 min. Measurements using H_2_O in place of ATP as well as reactions with buffer instead of protein served as blank and were subtracted from the ATP-data.

### Recql4 ATPase assay

PiColorLock phosphate detection reagent (Expedeon) was used to measure the presence of inorganic phosphate (Pi) release from ATP as a measure Recql4 ATPase activity. The proteins were assayed at 115nM in the presence of 1mM ATP and DNA in assay buffer (20mM HEPES pH 8.0, 10mM NaCl, 5% Glycerol, 1mM MgCl_2_, 0.5mM TCEP). Color change was measured at Abs_650nm_ in an EnSpire 2300 microplate reader (Perkin Elmer) at 25°C.

### Recql4 DNA binding assay

Electro-mobility shift assay (EMSA) was used to measure the relative binding of WT versus K525A mutant Recql4 to DNA binding. Protein was serially diluted from 300nM to 19nM and bound to 25nM single stranded DNA oligo XOm1 conjugated to IRDye680. Bound Protein-DNA complex was separated on a 6% TBE/Acrylamide gel. The gel was directly imaged on a Li-Cor Odyssey CLx near-infrared fluorescence imaging system (Millennium Science).

### Mice

*Recql4*^*K525A*^ and *Recql4*^*G522Efs*^ mice were generated using Cripsr/Cas9 methods by the Mouse Engineering at Garvan/ABR (MEGA) services (Garvan Institute, Darlinghurst, Australia). Lysine 525 was mutated to Alanine (AAG>GCA) in single cell C57Bl/6 embryos via sgRNA-directed gene targeting and homologous recombination with a single stranded DNA oligonucleotide substrate. Viable pups were screened by DNA sequencing and one C57Bl/6 male carrying the K525A mutation on one allele and a 2bp insertion (GA) after the T521 codon (G522Efs) on the other allele was identified as a founder. The chemically (ENU) induced *Recql4*^*M789K*^ and *Recql4*^*R347X*^ mutations were obtained from the Australian Phenomics Facility (APF, Canberra, Australia: IGL01381 and IGL01809). *Recql4*^*fl/fl*^ mice (C57BL/6-*Recql4*^*tm2272Arte*^) have been previously described [20, 44]. *Rosa26*-CreER^T2^ mice on a C57Bl/6 background were purchased from The Jackson Laboratory (B6.129-*Gt(ROSA)26Sor*^*tm1(cre/ERT2)Tyj*^/J, Stock Number: 008463) and have been previously described [20]. All lines were on a backcrossed C57Bl/6 background. ENU mutants were outcrossed at least 6 times and assessed across multiple generations to eliminate effects of any additional mutations. Tamoxifen containing food was prepared by Specialty Feeds (Perth, Australia) at 400mg/kg tamoxifen citrate (Sigma Aldrich) in a base of standard mouse chow.

### Genotyping

Genotyping of the K525A mutants was performed by PCR using the following primers: mRecql4 K525A MO36-F9: 5’-TAGACCTTATGAAACCTCAAAGCC-3’ and mRecql4 K525A MO36-R3: 5’- AGAACATTGGGCATTCGGC-3’ to yield a 591bp product, which was then digested with MslI (NEB) restriction enzyme to generate two fragments of 416 and 175bp for the WT or three fragments of 361, 175 and 55bp for the K525A mutant. Primers for the M789K mutants are: mRecql4 M789K 1F: 5’- AATAGGTGGCAATGGGCAGG-3’ and: mRecql4 M789K 1R: 5’-GCACTCGGCGAAAGGATACA-3’ yielding a 420bp PCR product, uncut by MslI when M789K mutant, but cut in two (277 and 143bp) when WT. The presence of the G522Efs and R347X mutations was determined by KASP (competitive allele specific PCR) technology (LGC) with custom designed (G522Efs) or facility provided (R347X primer: 5’- GAAGGTGACCAAGTTCATGCTAAAGCGTTTGTTTTTCATGTTGAGTCG-3’, 5’- GAAGGTCGGAGTCAACGGATTCAAAGCGTTTGTTTTTCATGTTGAGTCA-3’, reverse primer 5’-GCTTCCCTAGACAGAGGGAACTATA-3’) sequences according to manufacturer instructions.

### Protein extraction and western blotting

Thymocyte lysates were prepared in RIPA buffer (50mM Tris, 150mM NaCl, 1% NP-40, 0.5% sodium deoxycholate, 0.1% SDS, pH8.0) plus Complete protease inhibitor (Roche, 04693116001) and PhosStop (Roche, 4906837001) tablets. 25μg whole protein extracts were fractionated on pre-cast NuPAGE BOLT 8% Bis-Tris polyacrylamide gels (Invitrogen) and transferred onto Immobilon-P PVDF membranes (Merck Millipore). Membranes were blocked with 5% milk in TBST and incubated overnight with rat monoclonal anti-mouse Recql4 antibody (clone 3B10, WEHI Antibody Services, Walter and Eliza Hall Institute Biotechnology Centre) or mouse anti-β-Actin (Sigma Aldrich, A1978). Membranes were then probed with HRP-conjugated goat anti-rat (Thermo Fisher Scientific, 31470) or anti-mouse (Thermo Fisher Scientific, 31444) secondary antibodies and visualized using ECL Prime Substrate (Amersham).

### Peripheral blood analysis

Peripheral blood was analyzed on a hematological analyzer (Sysmex KX-21N, Roche Diagnostics). For flow cytometric analysis, red blood cells were lysed using a red blood cell lysis buffer (150mM NH_4_Cl, 10mM KHCO_3_, 0.1mM Na_2_EDTA, pH7.3).

### Flow cytometry analysis

Bones were flushed, spleens and thymus crushed, and single cell suspensions were prepared in PBS containing 2% FBS. Antibodies against murine Ter119, CD71, B220, IgM, CD43, CD19, CD21, CD23, Mac-1, Gr1, F4/80, CD4, CD8, TCRβ, CD25, CD44, Sca-1, c-Kit, CD34, FLT3, FcγRII/III (CD16/32), CD41, CD105, CD150, either biotinylated or conjugated with phycoerythrin, phycoerythrin-Cy7, peridinin chlorophyll protein-Cy5.5, allophycocyanin, allophycocyanin eFluor780, eFluor660 or eFluor450 were all obtained from eBioscience, BioLegend or BD Pharmingen (S1 Table) [20, 45, 46]. Biotinylated antibodies were detected with streptavidin conjugated with Brilliant Violet-605. 30,000–500,000 cells were acquired on a BD LSRIIFortessa and analyzed with FlowJo software Version 9 or 10.0 (Treestar).

### *In vitro* class switch recombination assay

B cells were purified from single cell spleen suspensions using a B Cell Isolation kit (Miltenyi, 130-090-862); 10^6^ cells per well were cultured in 6-well plates for 4 days in RPMI supplemented with 10% FCS, 100U/ml penicillin, 100ng/ml streptomycin, 2mM L-glutamine, 1 x MEM nonessential amino acids, 1mM sodium pyruvate, 50μM ß-mercaptoethanol, 15μg/ml LPS (Invivogen, tlrl-3pelps) and 10ng/ml recombinant murine IL-4 (Peprotech, 214–14), and stained with CellTrace Violet (Thermo Fisher Scientific, C34557) and rat anti-mouse IgG1-APC (BD Pharmingen, 550874) [47]. Stained cells were assessed using a LSRIIFortessa (BD) and data analysed using FACSDiva (BD) or FlowJo (Tree Star) software.

### *In vitro* DNA damage assay

Hoxb8 immortalized [23] *R26*-CreER^T2^ *Recql4*^*fl/+*^ (control) and *R26*-CreER^T2^ *Recql4*^*fl/K525A*^ cells were maintained in IMDM, 10% FBS (non-heat inactivated) and 1% GM-CSF containing media (BHK-HM5 cell conditioned media). The cells were treated for 4 days with 400nM 4-hydroxy tamoxifen (Merck Millipore) then genotyped to confirm complete recombination. Cells were then plated at 10,000 cells/well in 96 well plates (Corning, CLS3610) and incubated for 48 hours with the indicated concentration of drugs in triplicates per dose (dose range Doxorubicin: 0–0.5μM, Hydroxyurea: 0–0.5mM, 4-Nitroquinoline: 0–2μM and Topotecan: 0–0.5mM). Doxorubicin was obtained from St. Vincent’s Hospital Pharmacy. Hydroxyurea was purchased from Selleck. 4-Nitroquinoline and Topotecan were purchased from Sigma-Aldrich. Cell viability was measured using ATP-Lite (Perkin Elmer) as directed by the manufacturer and measured on an EnSpire plate reader (Perkin Elmer). Data were plotted and the IC_50_ value calculated using Prism 7 software. The dose-response curve was plotted as mean±SEM.

## Supporting information

S1 Fig Analysis of recombination and genotyping of point mutant alleles. Genomic PCR showing recombination of the floxed allele to produce the excised product of the expected size (left) and genotyping PCRs of the point mutants (right) of the following alleles: (A) M789K. (B) K525A. (C) G522Efs. (D) R347*.
(TIF)

S2 Fig *RECQL4* mutations in COSMIC database (110 mutations; somatic mutations). Data from COSMIC database. Image generated using Protein painter (Pecan portal St Jude’s).
(TIF)

S3 Fig Drug assays—individual doses dataset. Data showing the response to each individual drug dose used to calculate the IC_50_ values in Fig 5 from *R26*-CreER *Recql4*^*Δ/K525A*^ and *R26*-CreER *Recql4*^*Δ/+*^ with the non-tamoxifen treated isogenic controls. The X axis shows the individual drug doses of (A) Doxorubicin, (B) Hydroxyurea, (C) 4-Nitroquinoline, (D) Topotecan.
(TIF)

S1 Table FACS antibodies (anti-mouse). List of antibodies used for flow cytometry in this study.
(PDF)
