# Supplementary figures and images for: ATP-dependent helicase activity is dispensable for the physiological functions of Recql4
Source: PLoS Genet. 2019 Jul 5;15(7):e1008266. doi: 10.1371/journal.pgen.1008266 (PMC6636780; doi:10.1371/journal.pgen.1008266)

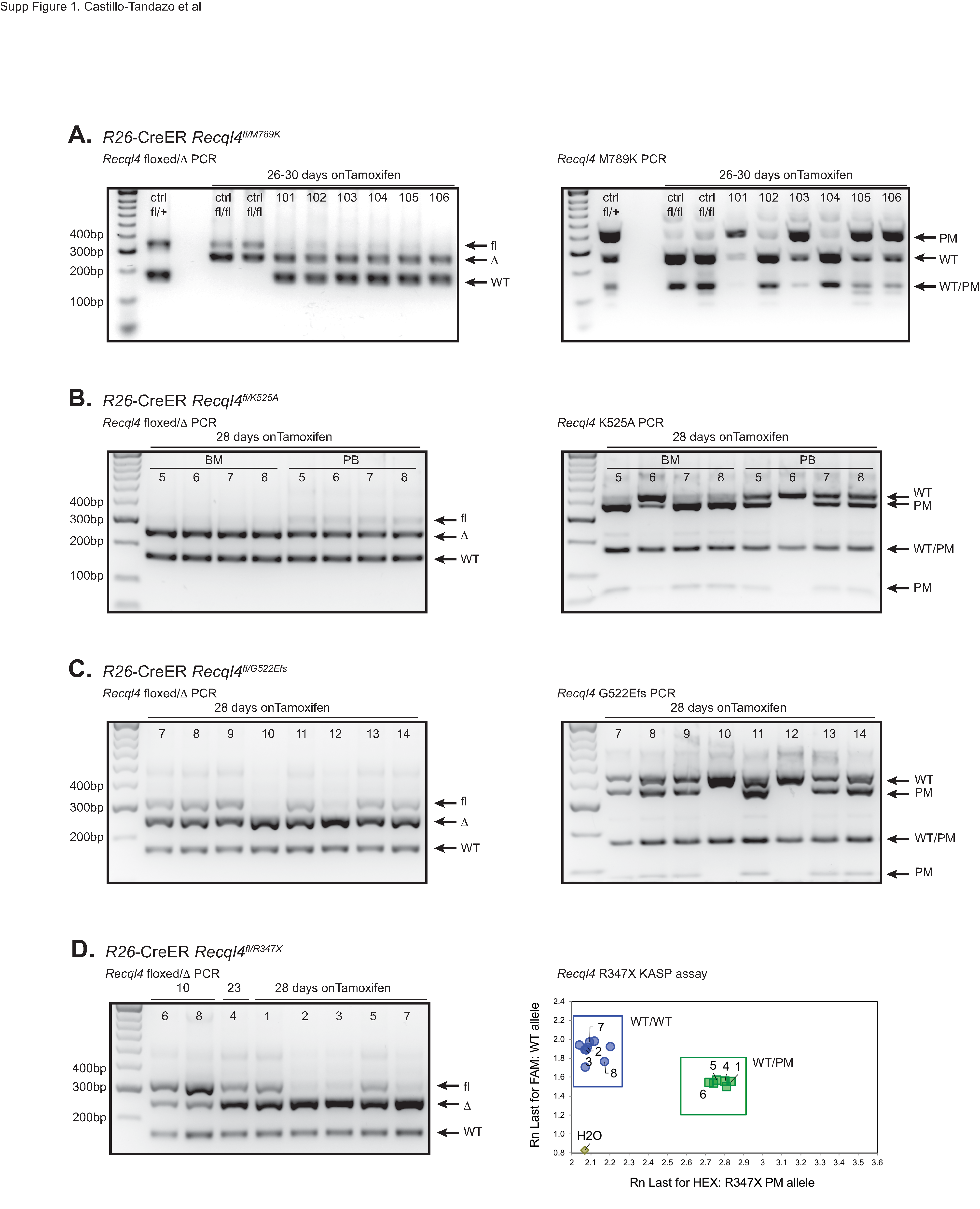

Supplement: S1 Fig — Genomic PCR showing recombination of the floxed allele to produce the excised product of the expected size (left) and genotyping PCRs of the point mutants (right) of the following alleles: (A) M789K. (B) K525A. (C) G522Efs. (D) R347*. (TIF) [file pgen.1008266.s001.tif]

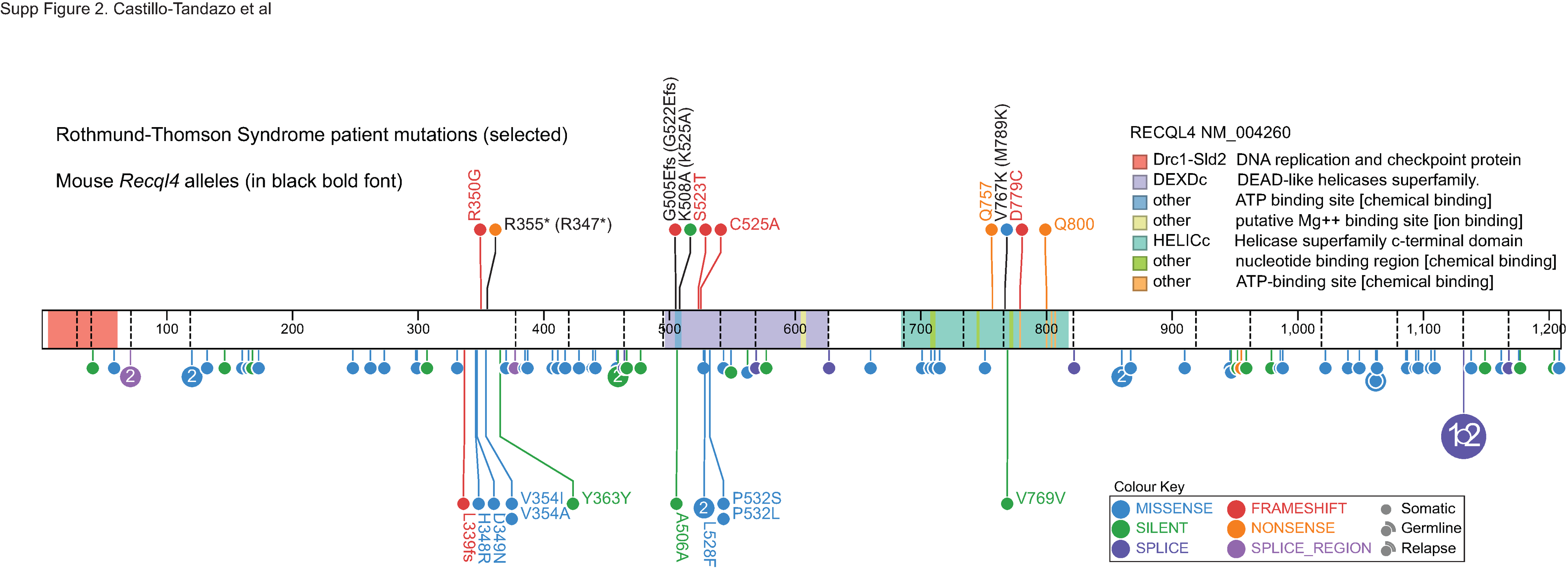

Supplement: S2 Fig — Data from COSMIC database. Image generated using Protein painter (Pecan portal St Jude’s). (TIF) [file pgen.1008266.s002.tif]

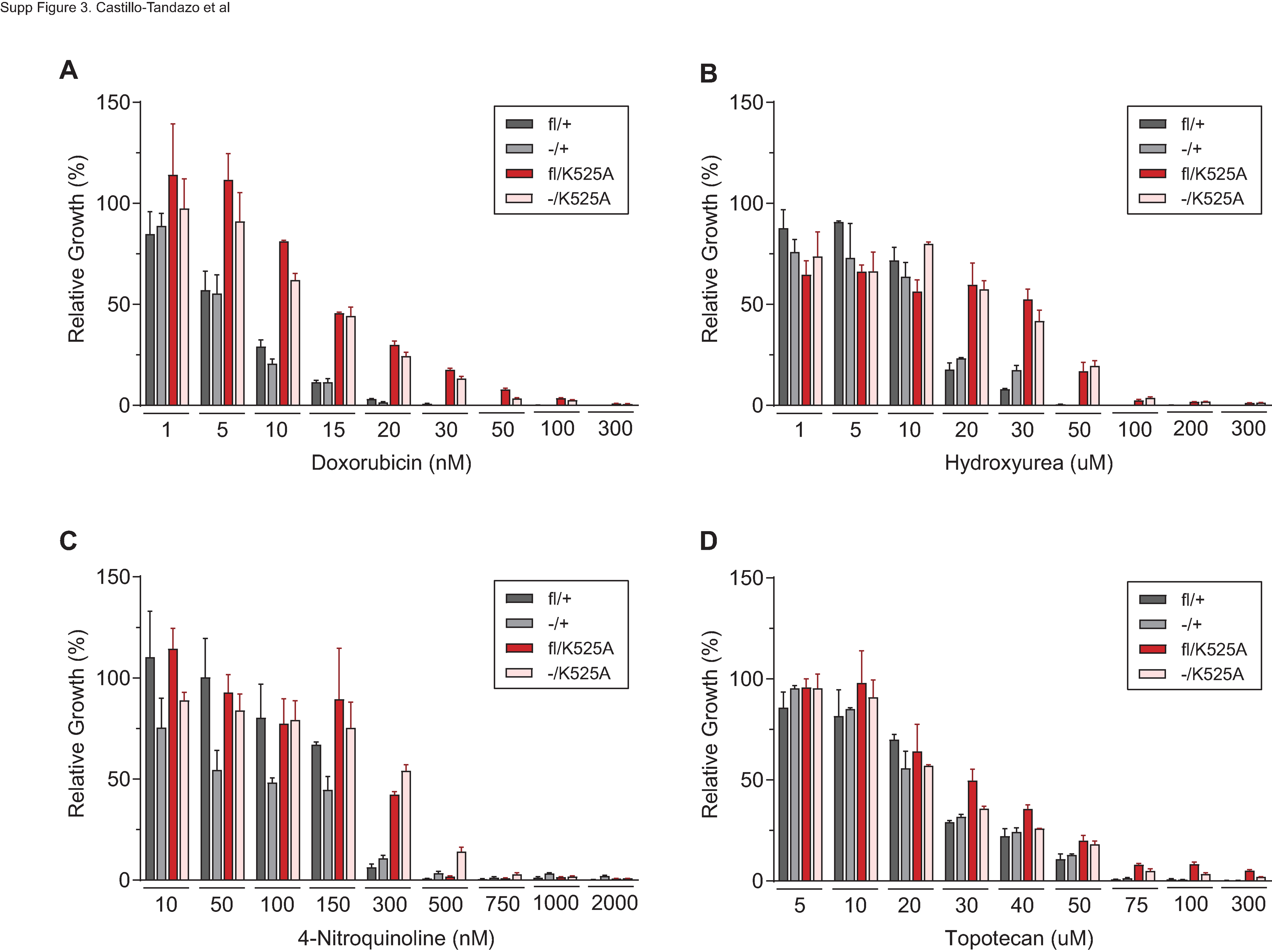

Supplement: S3 Fig — Data showing the response to each individual drug dose used to calculate the IC50 values in Fig 5 from R26-CreER Recql4Δ/K525A and R26-CreER Recql4Δ/+ with the non-tamoxifen treated isogenic controls. The X axis shows the individual drug doses of (A) Doxorubicin, (B) Hydroxyurea, (C) 4-Nitroquinoline, (D) Topotecan. (TIF) [file pgen.1008266.s003.tif]
