# Supplementary material for: ATP-dependent helicase activity is dispensable for the physiological functions of Recql4
Source: PLoS Genet. 2019 Jul 5;15(7):e1008266. doi: 10.1371/journal.pgen.1008266 (PMC6636780; doi:10.1371/journal.pgen.1008266)
Supplement: S1 Table — List of antibodies used for flow cytometry in this study. (PDF) [file pgen.1008266.s004.pdf]

S1 Table. FACS Antibodies (anti-mouse)

Antibody (clone)	Conjugate	Catalogue #	Company
Ter119 (TER-119)	PE	12-5921-83	Life Technologies Australia Pty Ltd/Thermo Fisher
CD71 (RI7 217.1.4)	APC	17-0711-82	Life Technologies Australia Pty Ltd/Thermo Fisher
B220/CD45R (RA3-6B2)	APC	17-0452-83	Life Technologies Australia Pty Ltd/Thermo Fisher
IgM (II/41)	Biotin	13-5790-82	Life Technologies Australia Pty Ltd/Thermo Fisher
CD43 (S7)	PE	553271	BD Pharmingen
CD19 (1D3)	PerCP-Cy5.5	45-0193-82	Life Technologies Australia Pty Ltd/Thermo Fisher
Mac1/CD11b (M1/70)	PE	12-0112-83	Life Technologies Australia Pty Ltd/Thermo Fisher
Gr-1/Ly6G (RB6-8C5)	PE-Cy7	25-5931-82	Life Technologies Australia Pty Ltd/Thermo Fisher
F4/80 (BM8.1)	APC	20-4801-U100	Tonbo Biosciences
CD4 (RM4-5)	eFluor-450	48-0042-82	Life Technologies Australia Pty Ltd/Thermo Fisher
CD8a/Ly-2 (53-6.7)	APC-eFluor 780	47-0081-82	Life Technologies Australia Pty Ltd/Thermo Fisher
TCRb (H57-597)	PE	12-5961-83	Life Technologies Australia Pty Ltd/Thermo Fisher
CD25 (PC61.5)	PE	12-0251-83	Life Technologies Australia Pty Ltd/Thermo Fisher
CD44 (IM7)	APC	17-0441-83	Life Technologies Australia Pty Ltd/Thermo Fisher
Sca-1 (D7)	APC	17-5981-82	Life Technologies Australia Pty Ltd/Thermo Fisher
c-Kit/CD117 (2B8)	APC-eFluor-780	47-1171-82	Life Technologies Australia Pty Ltd/Thermo Fisher
CD34 (RAM34)	eFluor-660	50-0341-82	Life Technologies Australia Pty Ltd/Thermo Fisher
CD135/Flk2/Flt3 (A2F10)	PE	12-1351-82	Life Technologies Australia Pty Ltd/Thermo Fisher
FcgR/CD16/32 (93)	PerCP-Cy5.5	45-0161-82	Life Technologies Australia Pty Ltd/Thermo Fisher
CD41 (MWReg30)	eFluor-450	48-0411-82	Life Technologies Australia Pty Ltd/Thermo Fisher
CD105 (MJ7/18)	PE-Cy7	120409	Biolegend
CD150 (TC15-12F12.2)	PE	115904	Biolegend
CD23	Biotin*	553137	BD Pharmingen
CD21	PE	123410	Biolegend
Streptavidin	BV 605	563260	BD Pharmingen
*Streptavidin (used with CD23)	BV 605	405229	Biolegend
